# Supplementary material for: Digital inequalities in health information seeking behaviors and experiences in the age of web 2.0: A population-based study in Hong Kong
Source: PLoS One. 2021 Mar 30;16(3):e0249400. doi: 10.1371/journal.pone.0249400 (PMC8009409; doi:10.1371/journal.pone.0249400)
Supplement: S1 File — (PDF) [file pone.0249400.s001.pdf]

Chinese version of the Information Seeking Experience (ISEE) Scale

Based on your latest search for health or medical information from the Internet, how much do you agree with the following statements? [Read out each option]
根據你最近一次用互聯網搵健康或醫學資訊嘅經驗，你有幾同意以下講法呢？[讀出各項]

	Very much agree 十分同意	Somewhat agree 部份同意	Somewhat disagree 唔太同意	Very much disagree 十分唔同意	Never used this source 冇使用	Refuse to answer 拒絕回答
a. It took a lot of effort to get the information you needed 你需要花好多心機先可以搵到你要嘅資訊	1	2	3	4	5	-99
b. You felt frustrated during your search for the information 你係搵所需資訊嘅過程中感到挫折	1	2	3	4	5	-99
c. You were concerned about the quality of the information 你擔心搵到嘅資訊質素	1	2	3	4	5	-99
d. The information you found was too hard to understand 你覺得搵到嘅資訊難以明白	1	2	3	4	5	-99
