# Supplementary material for: Digital inequalities in health information seeking behaviors and experiences in the age of web 2.0: A population-based study in Hong Kong
Source: PLoS One. 2021 Mar 30;16(3):e0249400. doi: 10.1371/journal.pone.0249400 (PMC8009409; doi:10.1371/journal.pone.0249400)
Supplement: S2 File — (PDF) [file pone.0249400.s002.pdf]

Chinese version of the two-item Patient Health Questionnaire (PHQ-2)

In the past 2 weeks, how often are you troubled by the following problems? [Read out each option]
過去兩個星期，你有幾經常受到以下問題困擾？[讀出各項]

	Never<br>完全沒有	Less than 7 days<br>少於 7 日	7 days or more<br>7 日或以上	Nearly daily<br>近乎每日	Refuse to answer<br>拒絕回答
a. Feel tired of doing anything, or don’t even want to do anything at all.<br>做任何事都覺得沉悶或者根本唔想做任何事	0	1	2	3	-99
b. Feel downhearted, depressed or desperate.<br>情緒低落、抑鬱或者絕望	0	1	2	3	-99
