# Supplementary material for: Digital inequalities in health information seeking behaviors and experiences in the age of web 2.0: A population-based study in Hong Kong
Source: PLoS One. 2021 Mar 30;16(3):e0249400. doi: 10.1371/journal.pone.0249400 (PMC8009409; doi:10.1371/journal.pone.0249400)
Supplement: S3 File — (DOCX) [file pone.0249400.s003.docx]

**Adjusted ^a^ associations of sociodemographic and health-related characteristics with health information seeking behaviors ^b^ using social networking sites, and instant messaging compared with internet websites**

|  | **Adjusted relative risk ratio (95% CI)** | |
| --- | --- | --- |
|  | **Social networking sites vs internet websites** | **Instant messaging vs internet websites** |
| **Sex** |  |  |
| Male | 1 | 1 |
| Female | 1.03 (0.73, 1.45) | 1.54 (1.14, 2.09)** |
| **Age, years** |  |  |
| 18–24 | 1 | 1 |
| 25–44 | 1.28 (0.63, 2.59) | 0.79 (0.28, 2.20) |
| 45–64 | 1.15 (0.54, 2.47) | 3.06 (1.12, 8.32)* |
| ≥65 | 1.76 (0.69, 4.47) | 7.73 (2.68, 22.26)*** |
| *P* for trend | 0.42 | <0.001 |
| **Marital status** |  |  |
| Never married | 1 | 1 |
| Divorced/separated/widowed | 1.32 (0.63, 2.76) | 2.64 (1.39, 5.02)** |
| Cohabitated/married | 1.02 (0.63, 1.63) | 2.48 (1.47, 4.18)** |
| **Educational attainment** |  |  |
| Primary or below | 1 | 1 |
| Secondary | 0.50 (0.26, 0.91)* | 0.37 (0.25, 0.57)*** |
| Tertiary | 0.19 (0.10, 0.37)*** | 0.14 (0.09, 0.23)*** |
| *P* for trend | <0.001 | <0.001 |
| **Employment status** |  |  |
| In-paid employed | 1 | 1 |
| Unemployed | 0.44 (0.15, 1.26) | 0.84 (0.37, 1.92) |
| Retired | 0.95 (0.55, 1.65) | 1.14 (0.79, 1.66) |
| Housekeeper | 0.54 (0.31, 0.94)* | 0.78 (0.53, 1.14) |
| Full-time student | 1.22 (0.57, 2.60) | 0.81 (0.23, 2.85) |
| **Monthly household income (HK $) ^c^** |  |  |
| ≤9999 | 1 | 1 |
| 10000­–19999 | 0.60 (0.32, 1.13) | 1.17 (0.73, 1.87) |
| 20000­–29999 | 0.82 (0.45, 1.49) | 1.06 (0.66, 1.70) |
| 30000­–39999 | 0.46 (0.24, 0.90) | 0.75 (0.45, 1.24) |
| ≥40000 | 0.49 (0.27, 0.89) | 0.68 (0.43, 1.07) |
| *P* for trend | 0.01 | 0.01 |
| Unstable or refused | 0.91 (0.48, 1.73) | 1.03 (0.61, 1.73) |
| **Smoking Status** |  |  |
| Never | 1 | 1 |
| Ex-smoker | 1.23 (0.70, 2.16) | 1.08 (0.65, 1.78) |
| Current smoker | 1.20 (0.68, 2.11) | 0.71 (0.44, 1.15) |
| **Alcohol drinking** |  |  |
| Never | 1 | 1 |
| Ex-drinker | 1.93 (0.91, 4.11) | 0.97 (0.48, 1.93) |
| Occasional drinker | 0.85 (0.59, 1.23) | 0.63 (0.47, 0.84)** |
| Less than once a month | 0.83 (0.45, 1.54) | 0.70 (0.40, 1.21) |
| 1 day/week or more | 0.97 (0.54, 1.76) | 0.81 (0.51, 1.31) |
| **Moderate physical activity** |  |  |
| None | 1 | 1 |
| 1–3 days/week | 1.21 (0.84, 1.74) | 0.78 (0.56, 1.08) |
| 4 days/week or more | 0.94 (0.61, 1.44) | 1.05 (0.77, 1.44) |
| **Diagnosed chronic diseases** |  |  |
| No | 1 | 1 |
| Yes | 1.72 (1.20, 2.46)** | 1.16 (0.88, 1.53) |
| **Screening for depression symptoms** |  |  |
| Negative (PHQ-2<3) | 1 | 1 |
| Positive (PHQ-2≥3) | 0.88 (0.48, 1.62) | 0.83 (0.48, 1.44) |

CI, Confidence Interval; PHQ-2, Patient Health Questionnaire-2 Item, range 0–6; **P**<*0.05, ***P<*0.01, ****P<*0.001.

^a^ Adjusted for sex, age, marital status, educational attainment, employment status, monthly household income, survey phase, and survey frame.

^b^ Frequency of health information seeking behavior was treated as a dummy variable (1= “at least once a week/1–3 times in a month/once in several months” vs 0= “seldom/never”). Respondents reporting seldom/never used the three sources or used multiple sources were excluded.

^c^ US $1 = HK $7.8
