# Supplementary material for: Digital inequalities in health information seeking behaviors and experiences in the age of web 2.0: A population-based study in Hong Kong
Source: PLoS One. 2021 Mar 30;16(3):e0249400. doi: 10.1371/journal.pone.0249400 (PMC8009409; doi:10.1371/journal.pone.0249400)
Supplement: S4 File — (DOCX) [file pone.0249400.s004.docx]

**Adjusted ^a^ associations of sociodemographic and health-related characteristics with web-based health information seeking experiences ^b^**

|  | **Adjusted odds ratios (95% CI)** | | | |
| --- | --- | --- | --- | --- |
|  | **It took a lot of effort to get the information you needed (n=3530)** | **You felt frustrated**  **during your search for the information (n=3506)** | **The information you found was too hard to understand ( n=3560)** | **You were concerned about the quality of the information (n=3546)** |
| **Sex** |  |  |  |  |
| Male | 1 | 1 | 1 | 1 |
| Female | 0.87 (0.76, 0.99)* | 0.97 (0.85, 1.11) | 0.94 (0.82, 1.08) | 0.93 (0.81, 1.06) |
| **Age, years** |  |  |  |  |
| 18–24 | 1 | 1 | 1 | 1 |
| 25–44 | 1.07 (0.83, 1.37) | 1.02 (0.79, 1.32) | 1.32 (1.02, 1.72)* | 1.07 (0.83, 1.39) |
| 45–64 | 1.56 (0.87, 1.53) | 1.23 (0.92, 1.64) | 1.53 (1.14, 2.04)** | 0.86 (0.64, 1.15) |
| ≥65 | 1.11 (0.75, 1.64) | 1.13 (0.76, 1.70) | 1.45 (0.97, 2.16) | 0.63 (0.42, 0.94)* |
| *P* for trend | 0.36 | 0.11 | 0.19 | 0.007 |
| **Marital status** |  |  |  |  |
| Never married | 1 | 1 | 1 | 1 |
| Divorced/ separated/ widowed | 1.02 (0.73, 1.42) | 1.08 (0.76, 1.52) | 1.01 (0.72, 1.41) | 1.06 (0.76, 1.48) |
| Cohabitated/ married | 1.01 (0.73, 1.42) | 1.17 (0.76, 1.52) | 1.02 (0.84, 1.22) | 0.91 (0.76, 1.10) |
| **Educational attainment** |  |  |  |  |
| Primary or below | 1 | 1 | 1 | 1 |
| Secondary | 0.58 (0.41, 0.80)** | 0.54 (0.38, 0.77)** | 0.45 (0.32, 0.63)*** | 1.12 (0.80, 1.58) |
| Tertiary | 0.47 (0.34, 0.67)*** | 0.43 (0.30, 0.62)*** | 0.33 (0.23, 0.47)*** | 1.22 (0.85, 1.73) |
| *P* for trend | <0.001 | <0.001 | <0.001 | 0.19 |
| **Employment status** |  |  |  |  |
| In-paid employed | 1 | 1 | 1 | 1 |
| Unemployed | 0.95 (0.65, 1.39) | 0.97 (0.66, 1.42) | 0.97 (0.66, 1.42) | 0.97 (0.66, 1.43) |
| Retired | 1.00 (0.78, 1.28) | 1.47 (1.14, 1.90)** | 1.01 (0.78, 1.31) | 1.26 (0.97, 1.62) |
| Housekeeper | 1.24 (1.00, 1.55) | 1.44 (1.15, 1.80)** | 1.10 (0.88, 1.37) | 1.13 (0.91, 1.42) |
| Full-time student | 0.67 (0.51, 0.89) | 0.84 (0.63, 1.11) | 1.02 (0.77, 1.36) | 1.23 (0.93, 1.63) |
| **Monthly household income (HK $) ^c^** |  |  |  |  |
| ≤9999 | 1 | 1 | 1 | 1 |
| 10000­–19999 | 0.96 (0.72, 1.29) | 1.08 (0.80, 1.46) | 0.87 (0.65, 1.17) | 1.10 (0.82, 1.48) |
| 20000­–29999 | 0.93 (0.70, 1.25) | 1.02 (0.76, 1.37) | 0.80 (0.60, 1.07) | 1.25 (0.94, 1.66) |
| 30000­–39999 | 0.96 (0.71, 1.28) | 0.96 (0.71, 1.29) | 0.76 (0.57, 1.02) | 1.12 (0.84, 1.50) |
| ≥40000 | 0.73 (0.55, 0.96)* | 0.76 (0.57, 1.01) | 0.70 (0.53, 0.91)** | 1.04 (0.79, 1.37) |
| *P* for trend | 0.004 | 0.001 | 0.006 | 0.73 |
| Unstable or refused | 0.76 (0.56, 1.04) | 0.93 (0.68, 1.28) | 0.80 (0.58, 1.09) | 0.89 (0.65, 1.21) |
| **Smoking Status** |  |  |  |  |
| Never | 1 | 1 | 1 | 1 |
| Ex-smoker | 0.91 (0.72, 1.16) | 0.80 (0.63, 1.03) | 0.96 (0.75, 1.23) | 0.93 (0.73, 1.18) |
| Current smoker | 1.03 (0.82, 1.29) | 0.92 (0.73, 1.17) | 1.10 (0.87, 1.39) | 1.05 (0.83, 1.32) |
| **Alcohol drinking** |  |  |  |  |
| Never | 1 | 1 | 1 | 1 |
| Ex-drinker | 0.96 (0.65, 1.43) | 0.95 (0.63, 1.42) | 0.71 (0.47, 1.08) | 0.74 (0.49, 1.13) |
| Occasional drinker | 0.91 (0.79, 1.05) | 0.95 (0.82, 1.10) | 1.10 (0.95, 1.27) | 1.08 (0.93, 1.25) |
| Less than once a month | 0.91 (0.72, 1.14) | 0.95 (0.76, 1.19) | 1.15 (0.91, 1.44) | 1.21 (0.96, 1.52) |
| 1 day/week or more | 0.83 (0.67, 1.03) | 0.82 (0.66, 1.03) | 0.98 (0.78, 1.22) | 0.95 (0.75, 1.19) |
| **Moderate physical activity** |  |  |  |  |
| None | 1 | 1 | 1 | 1 |
| 1–3 days/week | 1.12 (0.97, 1.29) | 1.06 (0.92, 1.23) | 1.04 (0.89, 1.20) | 0.98 (0.85, 1.40) |
| 4 days/week or more | 1.08 (0.92, 1.27) | 1.07 (0.91, 1.26) | 1.02 (0.86, 1.20) | 1.06 (0.90, 1.25) |
| **Diagnosed chronic diseases** |  |  |  |  |
| No | 1 | 1 | 1 | 1 |
| Yes | 1.03 (0.88, 1.21) | 1.10 (0.94, 1.29) | 1.00 (0.85, 1.18) | 1.10 (0.93, 1.29) |
| **Screening for depression symptoms** |  |  |  |  |
| Negative (PHQ-2<3) | 1 | 1 | 1 | 1 |
| Positive (PHQ-2≥3) | 1.25 (1.00, 1.57) | 1.24 (0.99, 1.57) | 1.18 (0.94, 1.49) | 1.25 (0.99, 1.57) |

CI, Confidence Interval; PHQ-2, Patient Health Questionnaire-2 Item, range 0–6; **P<*0.05, ***P<*0.01, ****P<*0.001.

^a^ Adjusted for sex, age, marital status, educational attainment, employment status, monthly household income, survey phase, and survey frame.

^b^ Agreement with web-based health information seeking experiences was treated as an ordinal variable (1=very much disagree, 2=somewhat disagree, 3=somewhat agree, 4=very much agree).

^c^ US $1 = HK $7.8
