# Supplementary material for: Digital inequalities in health information seeking behaviors and experiences in the age of web 2.0: A population-based study in Hong Kong
Source: PLoS One. 2021 Mar 30;16(3):e0249400. doi: 10.1371/journal.pone.0249400 (PMC8009409; doi:10.1371/journal.pone.0249400)
Supplement: Attachment — Submitted filename: Response to Reviewers.docx [file pone.0249400.s005.docx]

We are grateful to editors’ thoughtful comments. We have now revised the manuscript and provided point-to-point responses. Modifications in the text were also quoted in the responses below.

Additional Editor Comments:

Journal Requirements:

When submitting your revision, we need you to address these additional requirements.

Comment 1: Please ensure that your manuscript meets PLOS ONE's style requirements, including those for file naming. The PLOS ONE style templates can be found at

<https://journals.plos.org/plosone/s/file?id=wjVg/PLOSOne_formatting_sample_main_body.pdf> and

<https://journals.plos.org/plosone/s/file?id=ba62/PLOSOne_formatting_sample_title_authors_affiliations.pdf>

Response 1: Thank you for providing the helpful templates. We have now revised the manuscript format to meet PLOS ONE’s style requirements.

Comment 2: Please address the following:
- Please include additional information regarding the survey or questionnaire used in the study and ensure that you have provided sufficient details that others could replicate the analyses. For instance, if you developed a questionnaire as part of this study and it is not under a copyright more restrictive than CC-BY, please include a copy, in both the original language and English, as Supporting Information.

- Please provide additional details regarding participant consent. In the ethics statement in the Methods and online submission information, please ensure that you have specified how verbal consent was documented and witnessed).

Response 2:

- Thank you. We have now uploaded the Chinese version of the Information Seeking Experience (ISEE) Scale and the two-item Patient Health Questionnaire (PHQ-2) as Supporting Information 1 and Supporting Information 2, respectively. Details about other measures were shown in the Methods section.

- As suggested, in the ethics statement in the revised methods section and online submission information, we have now stated that “Verbal informed consent of all respondents was documented using the Web-CATI system under close supervision.”

We have now also added that “All data were collected by interviewers using a Web-based Computer Assisted Telephone Interview (Web-CATI) system invented in-house by the research team, which allowed real-time data capture and consolidation.” in the revised design and participants part.

Comment 3: We note that you have indicated that data from this study are available upon request. PLOS only allows data to be available upon request if there are legal or ethical restrictions on sharing data publicly. For information on unacceptable data access restrictions, please see <http://journals.plos.org/plosone/s/data-availability#loc-unacceptable-data-access-restrictions>.

In your revised cover letter, please address the following prompts:

a) If there are ethical or legal restrictions on sharing a de-identified data set, please explain them in detail (e.g., data contain potentially identifying or sensitive patient information) and who has imposed them (e.g., an ethics committee). Please also provide contact information for a data access committee, ethics committee, or other institutional body to which data requests may be sent.
b) If there are no restrictions, please upload the minimal anonymized data set necessary to replicate your study findings as either Supporting Information files or to a stable, public repository and provide us with the relevant URLs, DOIs, or accession numbers. Please see <http://www.bmj.com/content/340/bmj.c181.long> for guidelines on how to de-identify and prepare clinical data for publication. For a list of acceptable repositories, please see <http://journals.plos.org/plosone/s/data-availability#loc-recommended-repositories>.

We will update your Data Availability statement on your behalf to reflect the information you provide.

Response 3: In the revised cover letter, we have now stated that “The data underlying the findings of this study are restricted by the Institutional Review Board of the University of Hong Kong/Hospital Authority Hong Kong West, who approved the participant consent. Data requests can be sent to the FAMILY project (FAMILY: A Jockey Club Initiative for a Harmonious Society), G/F, Patrick Manson Building, 7 Sassoon Road, Pok Fu Lam, Hong Kong. Email: jcfamily@hku.hk.”
