# Supplementary material for: Digital inequalities in health information seeking behaviors and experiences in the age of web 2.0: A population-based study in Hong Kong
Source: PLoS One. 2021 Mar 30;16(3):e0249400. doi: 10.1371/journal.pone.0249400 (PMC8009409; doi:10.1371/journal.pone.0249400)
Supplement: Attachment — Submitted filename: R2_Plos_response_20200728.docx [file pone.0249400.s006.docx]

Comment 1: I find that you have clarified that the data is restricted by the IRB, and provided a contact method for data request. Could you also explain the restrictions in detail (e.g., data contain potentially identifying or sensitive patient information) in the statement as if what is described in the PLOS' data policy webpage.
<https://journals.plos.org/plosone/s/data-availability#loc-acceptable-data-sharing-methods>

Response 1: Thank you. We have now explained the restrictions in detail in the statement, as “The data underlying the findings of this study are restricted by the Institutional Review Board of the University of Hong Kong/Hospital Authority Hong Kong West, who approved the participant consent. Data contain potentially identifying information including direct identifiers (contact information) and indirect identifiers (location, occupation, income, etc.), which cannot be publicly shared in accordance with participant consent. Data requests can be sent to the FAMILY project (FAMILY: A Jockey Club Initiative for a Harmonious Society), G/F, Patrick Manson Building, 7 Sassoon Road, Pok Fu Lam, Hong Kong. Email: [jcfamily@hku.hk](mailto:jcfamily@hku.hk)”
