# Supplementary material for: Digital inequalities in health information seeking behaviors and experiences in the age of web 2.0: A population-based study in Hong Kong
Source: PLoS One. 2021 Mar 30;16(3):e0249400. doi: 10.1371/journal.pone.0249400 (PMC8009409; doi:10.1371/journal.pone.0249400)
Supplement: Attachment — Submitted filename: Response_HISB_20210106.docx [file pone.0249400.s007.docx]

**Reviewer 1**

Comment 1 (C1). This is a second review of this submission. The questions raised by the original reviewer have been answered properly. As a new reviewer in this process, I am in no position to raise new questions at this stage. On this basis, I recommend this submission as “accept”.

Response 1 (R1). Thank you for your comments.

**Reviewer 2**
C1. Some sentences in the conclusion (p.3 and p.24) are very confusing and inconsistent with your statistics results. Your results find that “lower educational attainment and income were associated with negative experiences regarding web-based seeking skills”, which suggests a digital divide between disadvantaged and advantaged groups. This is an important finding in this research, but it has contradicted the first sentence “Web 2.0 can reduce the physical barrier to HISBs and be promising health communication channels to reach the elderly and low-income groups” in the very beginning of the conclusion. That sentence could be a very brief interlude to the main conclusion, but it is inconsistent with the results. I suggest to drop or to re-write that inconsistent statement.

R1. We have now re-written the Conclusion as follows: “We identified correlates of web-based health information seeking and experiences in Hong Kong Chinese adults. Providing greater access to and improved information environment of web 2.0 to the target groups may help address digital inequalities.”

C2. Regarding Table 3, the author said “lower household income is associated with skill barriers” (p. 17). However, the adjusted prevalence ratios on Table 3 does not strongly support this argument. The author lists four web skill barriers measures. About their adjusted prevalence ratios, two are increasing with income levels and two are decreasing with income levels. The statement could be better revised as follows: the highest income group is associated with less skill barriers since the highest group has the lowest adjusted prevalence ratios on the three measures for sure.

R2. We originally stated that lower household income was associated with skill barriers because of inverse associations of income with feelings of effort and difficulties in understanding the information. We have now modified the statement in Results as follows: “Higher household income had decreased aPRs for feelings of effort (*P* for trend = 0.001; ≥ HK $40000: aPR = 0.84, 95% CI 0.72, 0.98) and difficulties in understanding the information (*P* for trend = 0.02).”

C3. Do you have only binomial answers (Y/N) to web experience questions? If not, Poisson regression might not be the best statistic model. You might need to try Cox or logistic regression to take account of ordinal or categorical dependent variables.

R3. The dichotomized responses to experiences (1 = very much agree/somewhat agree vs 0 = somewhat disagree/very much disagree) were consistent with studies using the same questions [1–3]. Poisson regression models fitted reasonably well from results of goodness-of-fit chi-squared tests and tests of equi-dispersion assumption (Please see R4 below).

We have now tested the robustness of our results using ordered logistic regression for ordinal responses: 1= very much disagree, 2 = somewhat disagree, 3 = somewhat agree, 4 = very much agree (Response Table 1). Similar associations are found between ordered logistic regression and Poisson regression. For example, lower educational attainment was associated with skill barriers, including feelings of effort, frustration, and difficulties in understanding the information.

**Response Table 1. Adjusted ^a^ associations of sociodemographic and health-related characteristics with web-based health information seeking experiences ^b^**

|  | **Adjusted odds ratios (95% CI)** | | | |
| --- | --- | --- | --- | --- |
|  | **It took a lot of effort to get the information you needed (n=3530)** | **You felt frustrated**  **during your search for the information (n=3506)** | **The information you found was too hard to understand ( n=3560)** | **You were concerned about the quality of the information (n=3546)** |
| **Sex** |  |  |  |  |
| Male | 1 | 1 | 1 | 1 |
| Female | 0.87 (0.76, 0.99)* | 0.97 (0.85, 1.11) | 0.94 (0.82, 1.08) | 0.93 (0.81, 1.06) |
| **Age, years** |  |  |  |  |
| 18–24 | 1 | 1 | 1 | 1 |
| 25–44 | 1.07 (0.83, 1.37) | 1.02 (0.79, 1.32) | 1.32 (1.02, 1.72)* | 1.07 (0.83, 1.39) |
| 45–64 | 1.56 (0.87, 1.53) | 1.23 (0.92, 1.64) | 1.53 (1.14, 2.04)** | 0.86 (0.64, 1.15) |
| ≥65 | 1.11 (0.75, 1.64) | 1.13 (0.76, 1.70) | 1.45 (0.97, 2.16) | 0.63 (0.42, 0.94)* |
| *P* for trend | 0.36 | 0.11 | 0.19 | 0.007 |
| **Marital status** |  |  |  |  |
| Never married | 1 | 1 | 1 | 1 |
| Divorced/ separated/ widowed | 1.02 (0.73, 1.42) | 1.08 (0.76, 1.52) | 1.01 (0.72, 1.41) | 1.06 (0.76, 1.48) |
| Cohabitated/ married | 1.01 (0.73, 1.42) | 1.17 (0.76, 1.52) | 1.02 (0.84, 1.22) | 0.91 (0.76, 1.10) |
| **Educational attainment** |  |  |  |  |
| Primary or below | 1 | 1 | 1 | 1 |
| Secondary | 0.58 (0.41, 0.80)** | 0.54 (0.38, 0.77)** | 0.45 (0.32, 0.63)*** | 1.12 (0.80, 1.58) |
| Tertiary | 0.47 (0.34, 0.67)*** | 0.43 (0.30, 0.62)*** | 0.33 (0.23, 0.47)*** | 1.22 (0.85, 1.73) |
| *P* for trend | <0.001 | <0.001 | <0.001 | 0.19 |
| **Employment status** |  |  |  |  |
| In-paid employed | 1 | 1 | 1 | 1 |
| Unemployed | 0.95 (0.65, 1.39) | 0.97 (0.66, 1.42) | 0.97 (0.66, 1.42) | 0.97 (0.66, 1.43) |
| Retired | 1.00 (0.78, 1.28) | 1.47 (1.14, 1.90)** | 1.01 (0.78, 1.31) | 1.26 (0.97, 1.62) |
| Housekeeper | 1.24 (1.00, 1.55) | 1.44 (1.15, 1.80)** | 1.10 (0.88, 1.37) | 1.13 (0.91, 1.42) |
| Full-time student | 0.67 (0.51, 0.89) | 0.84 (0.63, 1.11) | 1.02 (0.77, 1.36) | 1.23 (0.93, 1.63) |
| **Monthly household income (HK $) ^c^** |  |  |  |  |
| ≤9999 | 1 | 1 | 1 | 1 |
| 10000­–19999 | 0.96 (0.72, 1.29) | 1.08 (0.80, 1.46) | 0.87 (0.65, 1.17) | 1.10 (0.82, 1.48) |
| 20000­–29999 | 0.93 (0.70, 1.25) | 1.02 (0.76, 1.37) | 0.80 (0.60, 1.07) | 1.25 (0.94, 1.66) |
| 30000­–39999 | 0.96 (0.71, 1.28) | 0.96 (0.71, 1.29) | 0.76 (0.57, 1.02) | 1.12 (0.84, 1.50) |
| ≥40000 | 0.73 (0.55, 0.96)* | 0.76 (0.57, 1.01) | 0.70 (0.53, 0.91)** | 1.04 (0.79, 1.37) |
| *P* for trend | 0.004 | 0.001 | 0.006 | 0.73 |
| Unstable or refused | 0.76 (0.56, 1.04) | 0.93 (0.68, 1.28) | 0.80 (0.58, 1.09) | 0.89 (0.65, 1.21) |

CI, Confidence Interval; PHQ-2, Patient Health Questionnaire-2 Item, range 0–6; **P<*0.05, ***P<*0.01, ****P<*0.001.

^a^ Adjusted for sex, age, marital status, educational attainment, employment status, monthly household income, survey phase, and survey frame.

^b^ Agreement with web-based health information seeking experiences was treated as an ordinal variable (1=very much disagree, 2=somewhat disagree, 3=somewhat agree, 4=very much agree).

^c^ US $1 = HK $7.8

C4. Please provide goodness of fit statistics and perform diagnostic tests about your Poisson regressions. And briefly explain how you choose Poisson over log-binomial.

R4. The contents have been added in Methods as follows: “Note that log-binomial regression also estimates relative risk but is subject to narrower confidence intervals than they should be and convergence problems [4]. Stata’s “estat gof” command was used to yield goodness-of-fit statistics and the “nbreg” command was used to check the equi-dispersion assumption of Poisson regression. All Poisson regression models were supported as all goodness-of-fit chi-squared tests and tests of dispersion were found not statistically significant (all *P* = 1.00).”

**Suggestions:**
C5. Web 2.0 is a very general and old idea. Nowadays, we need more precise and detailed discussions about the web. For example, “Web information” in this research includes information from websites, social networks, and instant message. Characteristics of the three information sources are very different; the differences may lead to very divergent web behaviors and web experiences. It would be very meaningful to see the comparisons among the three groups regarding their web-based health information seeking experiences. In other words, I am interested in the relative risk among the three sources.

R5. We agree and have now compared experiences by the three different web-based sources in Methods as follows: “aPRs for experiences by different web-based sources adjusting for sociodemographic and health-related characteristics were yielded in respondents who exclusively used internet websites, SNS, or IM (at least once a week/1–3 times in a month/once in several months), whereas those seldom/never used the three sources or used multiple sources were excluded.” We have added Results as follows: “Quality concern was the most common negative web-based health information seeking experiences across different sources (74.5%–82.1%) (Table 5). Compared with internet websites, HISBs using IM was associated with feelings of frustration (aPR = 1.39, 95% CI 1.08, 1.79), difficulties in understanding the information (aPR = 1.36, 95% CI 1.12, 1.65), and being concerned about the qualities (aPR = 1.20, 95% CI 1.08, 1.32).” We have added Discussion as follows: “IM was associated with more negative experiences among the three web-based sources. Frustration and difficulties in understanding may be attributable to the lower readability of health information on IM as IM applications are designed with shorter text, smaller font size, and more crowded visual presentation than internet websites. Health information on IM may be from a small and closer social network not involving healthcare professionals. Nearly 70% of respondents were concerned about health information on WeChat in a national-wide survey in China [5]. Healthcare professionals can use WhatsApp Business or WeChat Official Account for delivering quality health information to the public.”

**Table 5. Weighted ^a^ n (%) and adjusted ^b^ prevalence ratios (aPRs) for web-based health information seeking experiences ^c^ by different sources**

|  | **It took a lot of effort to get the information you needed** | | | **You felt frustrated during your search for the information** | | | **The information you found was too hard to understand** | | | **You were concerned about the quality of the information** | | |
| --- | --- | --- | --- | --- | --- | --- | --- | --- | --- | --- | --- | --- |
|  | **Disagree, n (%)** | **Agree, n (%)** | **aPR (95% CI)** | **Disagree, n (%)** | **Agree, n (%)** | **aPR (95% CI)** | **Disagree, n (%)** | **Agree, n (%)** | **aPR (95% CI)** | **Disagree, n (%)** | **Agree, n (%)** | **aPR (95% CI)** |
| Internet websites | 646 (56.3) | 502 (43.7) | 1 | 864 (75.4) | 282 (24.6) | 1 | 695 (60.5) | 453 (39.5) | 1 | 291 (25.4) | 853 (74.6) | 1 |
| Social networking sites | 56 (66.8) | 28 (33.2) | 0.95 (0.71, 1.27) | 55 (62.9) | 32 (37.1) | 1.51 (1.11, 2.05)** | 45 (52.0) | 42 (48.0) | 1.24 (0.96, 1.60) | 22 (25.5) | 65 (74.5) | 1.01 (0.88, 1.16) |
| Instant messaging | 41 (40.0) | 62 (60.0) | 1.19 (0.98, 1.44) | 50 (48.4) | 53 (51.6) | 1.39 (1.08, 1.79)* | 38 (34.9) | 70 (65.1) | 1.36 (1.12, 1.65)** | 19 (18.0) | 88 (82.1) | 1.20 (1.08, 1.32)*** |

CI, Confidence Interval; **P*<0.05, ***P*<0.01, ****P*<0.001.

^a^ Weighted by sex, age, educational attainment according to Hong Kong Census.

^b^ Adjusted for sociodemographic and health-related characteristics, survey phase, and survey frame.

^c^ Agreement with web-based health information seeking experiences was treated as a dummy variable (Agree: 1= “very much agree/somewhat agree” vs Disagree: 0= “somewhat disagree/very much disagree”). Respondents reporting seldom/never used the three sources or used multiple sources were excluded.

C6. It is also important to ask whether the sociodemographic and health-related characteristics are associated with the choice among three different sources: websites, social networks and IM. Table 2 implies that the older groups (45-64; >64) and higher income groups (>= 10000) may rely more on IM. These results are worth further discussion since this paper is talking about “digital inequality”. However, you have better to run a regression regarding the three groups, rather than to compare the coefficients among three different regressions.

R6. We have now run one regression model and added results as supporting information in S3. File. Specifically, a multinomial logistic regression has been used to examine sociodemographic and health-related correlates of preferred web-based sources: SNS, IM, and internet websites (reference outcome). S3. File shows that older group was more likely to seek health information using IM compared with internet websites, which complemented our findings that IM may reduce digital inequalities for the older people in the original Table 2 (now Table 3). We have now enriched our statement in Discussion as follows: “Compared with internet websites (web 1.0), IM appeared to reduce digital inequalities in HISBs in older people in our study. Similar result that age was not a significant predictor of HISBs using web 2.0 was found in the United States [6]. Mobile phone for web 2.0 has higher penetration rate than personal computers for web 1.0 among the elderly in Hong Kong [7], possibly due to low-cost internet access and wide coverage of public free WiFi services (~ 51943 hotspots in 2017) [8]. Text messaging-based IM could be more popular due to the low requirements for technology skills. IM has been found as a feasible and effective intervention modality in promoting healthy behaviors in older people [9]…”

**S3. File.** **Adjusted ^a^ associations of sociodemographic and health-related characteristics with health information seeking behaviors ^b^ using social networking sites, and instant messaging compared with internet websites**

|  | **Adjusted relative risk ratio (95% CI)** | |
| --- | --- | --- |
|  | **Social networking sites vs internet websites** | **Instant messaging vs internet websites** |
| **Sex** |  |  |
| Male | 1 | 1 |
| Female | 1.03 (0.73, 1.45) | 1.54 (1.14, 2.09)** |
| **Age, years** |  |  |
| 18–24 | 1 | 1 |
| 25–44 | 1.28 (0.63, 2.59) | 0.79 (0.28, 2.20) |
| 45–64 | 1.15 (0.54, 2.47) | 3.06 (1.12, 8.32)* |
| ≥65 | 1.76 (0.69, 4.47) | 7.73 (2.68, 22.26)*** |
| *P* for trend | 0.42 | <0.001 |
| **Marital status** |  |  |
| Never married | 1 | 1 |
| Divorced/separated/widowed | 1.32 (0.63, 2.76) | 2.64 (1.39, 5.02)** |
| Cohabitated/married | 1.02 (0.63, 1.63) | 2.48 (1.47, 4.18)** |
| **Educational attainment** |  |  |
| Primary or below | 1 | 1 |
| Secondary | 0.50 (0.26, 0.91)* | 0.37 (0.25, 0.57)*** |
| Tertiary | 0.19 (0.10, 0.37)*** | 0.14 (0.09, 0.23)*** |
| *P* for trend | <0.001 | <0.001 |
| **Employment status** |  |  |
| In-paid employed | 1 | 1 |
| Unemployed | 0.44 (0.15, 1.26) | 0.84 (0.37, 1.92) |
| Retired | 0.95 (0.55, 1.65) | 1.14 (0.79, 1.66) |
| Housekeeper | 0.54 (0.31, 0.94)* | 0.78 (0.53, 1.14) |
| Full-time student | 1.22 (0.57, 2.60) | 0.81 (0.23, 2.85) |
| **Monthly household income (HK $) ^c^** |  |  |
| ≤9999 | 1 | 1 |
| 10000­–19999 | 0.60 (0.32, 1.13) | 1.17 (0.73, 1.87) |
| 20000­–29999 | 0.82 (0.45, 1.49) | 1.06 (0.66, 1.70) |
| 30000­–39999 | 0.46 (0.24, 0.90) | 0.75 (0.45, 1.24) |
| ≥40000 | 0.49 (0.27, 0.89) | 0.68 (0.43, 1.07) |
| *P* for trend | 0.01 | 0.01 |
| Unstable or refused | 0.91 (0.48, 1.73) | 1.03 (0.61, 1.73) |
| **Smoking Status** |  |  |
| Never | 1 | 1 |
| Ex-smoker | 1.23 (0.70, 2.16) | 1.08 (0.65, 1.78) |
| Current smoker | 1.20 (0.68, 2.11) | 0.71 (0.44, 1.15) |
| **Alcohol drinking** |  |  |
| Never | 1 | 1 |
| Ex-drinker | 1.93 (0.91, 4.11) | 0.97 (0.48, 1.93) |
| Occasional drinker | 0.85 (0.59, 1.23) | 0.63 (0.47, 0.84)** |
| Less than once a month | 0.83 (0.45, 1.54) | 0.70 (0.40, 1.21) |
| 1 day/week or more | 0.97 (0.54, 1.76) | 0.81 (0.51, 1.31) |
| **Moderate physical activity** |  |  |
| None | 1 | 1 |
| 1–3 days/week | 1.21 (0.84, 1.74) | 0.78 (0.56, 1.08) |
| 4 days/week or more | 0.94 (0.61, 1.44) | 1.05 (0.77, 1.44) |
| **Diagnosed chronic diseases** |  |  |
| No | 1 | 1 |
| Yes | 1.72 (1.20, 2.46)** | 1.16 (0.88, 1.53) |
| **Screening for depression symptoms** |  |  |
| Negative (PHQ-2<3) | 1 | 1 |
| Positive (PHQ-2≥3) | 0.88 (0.48, 1.62) | 0.83 (0.48, 1.44) |

CI, Confidence Interval; PHQ-2, Patient Health Questionnaire-2 Item, range 0–6; **P**<*0.05, ***P<*0.01, ****P<*0.001.

^a^ Adjusted for sex, age, marital status, educational attainment, employment status, monthly household income, survey phase, and survey frame.

^b^ Frequency of health information seeking behavior was treated as a dummy variable (1= “at least once a week/1–3 times in a month/once in several months” vs 0= “seldom/never”). Respondents reporting seldom/never used the three sources or used multiple sources were excluded.

^c^ US $1 = HK $7.8

**Reviewer 3**

C1. The authors chose four sources of health information, including the traditional source, internet websites, SNS (Facebook, Twitter), and IM (WeChat, WhatsApp). The authors are expected to justify the use of IM (WeChat, WhatsApp), since they are not apps from health-care agencies. Although the authors gave examples of the use of IM chat for the smoking cessation. It may merely serve research purposes.

R1. We have now elaborated on IM use in Introduction as follows: “For example, patients can share their experiences with healthcare providers, people with a similar medical issue, friends, or family members using IM [10]. WeChat group chat was one of the primary means of seeking health information in a national survey in China [5]. Other functions of IM can include online appointment scheduling and online medical consultation.”

C2. The research question seems too broad. For example, it targets the general health information without specifying the purpose the information seeking, such as disease prevention, self-monitoring of symptoms, health promotion, etc.

R2. The use of general health information was consistent with studies in the general population including ours [11] and others in the Western setting [12,13]. Nevertheless, we agree on the importance of purpose and have acknowledged the limitation in Discussion as follows: “We examined general health information seeking and experiences. Future studies are needed to differentiate the purpose, such as health promotion, disease prevention, treatment, or management.”

C3. The authors conducted two phases survey. Is there any trend in the two phases survey?

R3. Yes and we have now added a table on the trend in the two phases (Table 2) and described in Results as follows: “Prevalence of HISBs using all four sources increased from phase 1 to phase 2 (all *P* < 0.001).” and “Prevalence of agreeing that they were concerned about the quality (72.3% to 76.3%, *P* = 0.03) and that the information found was too hard to understand (40.6% to 49.0%, *P* < 0.001) increased from phase 1 to phase 2.”

**Table 2.** **Weighted ^a^ n (%) for health information seeking behavior and web-based health information seeking experiences by survey phases**

|  | **Total** | **Phase 1** | **Phase 2** | ***P*** |
| --- | --- | --- | --- | --- |
| **Health information seeking behaviors (at least once a week/1–3 times in a month/once in several months) ^b^** |  |  |  |  |
| Traditional sources (television, radio, newspaper, and magazine) (n = 10141) | 3585 (35.4) | 1658 (32.6) | 1927 (38.1) | <0.001 |
| Internet websites (n = 10138) | 3739 (36.9) | 1771 (34.9) | 1968 (38.9) | <0.001 |
| Social networking sites (n = 10140) | 1810 (17.9) | 818 (16.1) | 992 (19.6) | <0.001 |
| Instant messaging (n = 10140) | 1304 (12.9) | 581 (11.4) | 723 (14.3) | <0.001 |
| **Web-based health information seeking experiences (very much agree/somewhat agree) ^c^** |  |  |  |  |
| It took a lot of effort to get the information you needed (n = 3530) | 1638 (46.4) | 771 (45.6) | 866 (47.2) | 0.45 |
| You felt frustrated during your search for the information (n = 3506) | 1049 (29.8) | 491 (29.1) | 555 (30.5) | 0.50 |
| The information you found was too hard to understand (n = 3560) | 1601 (45.0) | 694 (40.6) | 907 (49.0) | <0.001 |
| You were concerned about the quality of the information (n = 3546) | 2637 (74.4) | 1233 (72.3) | 1404 (76.3) | 0.03 |

^a^ Weighted by sex, age, educational attainment according to Hong Kong Census.

^b^ Frequency of health information seeking behavior was treated as a dummy variable (1= “at least once a week/1–3 times in a month/once in several months” vs 0= “seldom/never”).

^c^ Agreement with web-based health information seeking experiences was treated as a dummy variable (1= “very much agree/somewhat agree” vs 0= “somewhat disagree/very much disagree”).

C4. The results showed that around 75% of the participants were concerned about the quality of the information. Thus, besides the access, confidence in the information plays an essential role in influencing people’s behavior. The authors are suggested to elaborate on this point in the Discussion section, for example, how to improve the quality of the information and how to influence people’s behavior.

R4. The point has now been elaborated in Discussion as follows: “Quality concern was the most common (74.4%) negative experience in our study. Such mental barrier may be due to the spread of health misinformation on web-based sources that allow the anonymity of content generator and disseminator and low rigor in monitoring and fact-checking [14] …Community-based interventions, such as collaborative learning and increased social support, may improve people’s confidence in dealing with the web-based information [15]. Healthcare professionals could leverage online platforms to disseminate evidence-based content, correct misinformation, and build trust with the communities. Technology companies can implement mechanisms for vetting and validating the credibility of information. For example, Twitter has now used labels and warning messages to add context and instructions on some Tweets containing disputed or misleading information [16].”

C5. The authors provided an overall picture of the information-seeking experience, such as concern about the quality of the information, frustration during the search, hard to understand the information, etc. It would be interesting to have a comparison among the different sources of HISB. For example, how many people complained the quality of information from traditional sources/internet websites/SNS/IM?

R5. We agree and have now added a table on comparing experiences by different sources (Table 5). Please see R5 to Reviewer 2 above.

**References**

1.	Vanderpool RC, Kornfeld J, Rutten LF, Squiers L. Cancer information-seeking experiences: the implications of Hispanic ethnicity and Spanish language. Journal of Cancer Education. 2009;24:141. doi:10.1080/08858190902854772

2.	Arora NK, Hesse BW, Rimer BK, Viswanath K, Clayman ML, Croyle RT. Frustrated and confused: the American public rates its cancer-related information-seeking experiences. Journal of General Internal Medicine. 2008;23: 223–228. doi:10.1007/s11606-007-0406-y

3.	Kim K, Lustria MLA, Burke D, Kwon N. Predictors of cancer information overload: findings from a national survey. Information research. 2007;12: 12–4.

4.	Zou G. A modified poisson regression approach to prospective studies with binary data. American Journal of Epidemiology. 2004;159: 702–706. doi:10.1093/aje/kwh090

5.	Zhang X, Wen D, Liang J, Lei J. How the public uses social media wechat to obtain health information in china: a survey study. BMC Medical Informatics and Decision Making. 2017;17. doi:10.1186/s12911-017-0470-0

6.	Tennant B, Stellefson M, Dodd V, Chaney B, Chaney D, Paige S, et al. eHealth literacy and web 2.0 health information seeking behaviors among baby boomers and older adults. Journal of Medical Internet Research. 2015;17: e70. doi:10.2196/jmir.3992

7.	Census and Statistics Department. Thematic Household Survey Report No. 69 - Personal computer and Internet penetration. 2020. Available: <https://www.ogcio.gov.hk/en/about_us/facts/doc/householdreport2020_69.pdf>

8.	Office of the Communications Authority. Public Wi-Fi Services. 2019. Available: <https://www.ofca.gov.hk/mobile/en/data_statistics/data_statistics/wifi/index.html>

9.	Kwan RY, Lee D, Lee PH, Tse M, Cheung DS, Thiamwong L, et al. Effects of an mHealth brisk walking intervention on increasing physical activity in older people with cognitive frailty: pilot randomized controlled trial. JMIR Mhealth Uhealth. 2020;8:e16596.. doi:10.2196/16596

10.	Iftikhar R, Abaalkhail B. Health-seeking influence reflected by online health-related messages received on social media: cross-sectional survey. Journal of Medical Internet Research. 2017;19: e382. doi:10.2196/jmir.5989

11.	Wang MP, Viswanath K, Lam TH, Wang X, Chan SS. Social determinants of health information seeking among Chinese Adults in Hong Kong. PloS one. 2013;8: e73049. doi:10.1371/journal.pone.0073049

12.	Jacobs W, Amuta AO, Jeon KC. Health information seeking in the digital age: an analysis of health information seeking behavior among US adults. Cogent Social Sciences. 2017;3. doi:10.1080/23311886.2017.1302785

13.	Cutrona SL, Mazor KM, Agunwamba AA, Valluri S, Wilson PM, Sadasivam RS, et al. Health information brokers in the general population: an analysis of the Health Information National Trends Survey 2013-2014. Journal of Medical Internet Research. 2016;18: e123. doi:10.2196/jmir.5447

14.	Chu JT, Wang MP, Shen C, Viswanath K, Lam TH, Chan SSC. How, when and why people seek health information online: qualitative study in Hong Kong. Interactive Journal of Medical Research. 2017;6: e24. doi:10.2196/ijmr.7000

15.	de Wit L, Fenenga C, Giammarchi C, di Furia L, Hutter I, de Winter A, et al. Community-based initiatives improving critical health literacy: a systematic review and meta-synthesis of qualitative evidence. BMC Public Health. 2017;18: 40. doi:10.1186/s12889-017-4570-7

16.	Roth Y, Pickles N. Updating our approach to misleading information. Available: https://blog.twitter.com/en_us/topics/product/2020/updating-our-approach-to-misleading-information.html
