# Supplementary material for: Digital inequalities in health information seeking behaviors and experiences in the age of web 2.0: A population-based study in Hong Kong
Source: PLoS One. 2021 Mar 30;16(3):e0249400. doi: 10.1371/journal.pone.0249400 (PMC8009409; doi:10.1371/journal.pone.0249400)
Supplement: Attachment — Submitted filename: Response_20210218.docx [file pone.0249400.s008.docx]

**Academic Editor's comments**

Comment 1 (C1). Tables: For the table with Poisson regression analysis, please also provide the corresponding Pseudo R-square for reference. This is for readers to have a better understanding of the models.

Response 1 (R1). We have now added Pseudo R-squares in Tables 3, 4 and 5.

C2. Ethics section: Please also provide the approval reference number.

R2. The approval reference number (UW 09-324) has now been provided in Ethics section.

C3. Others: Please add the data provision statement you provided as an author note at the end of your manuscript. Therefore, “The data underlying the findings of this study are restricted by the Institutional Review Board of the University of Hong Kong/Hospital Authority Hong Kong West, who approved the participant consent. Data contain potentially identifying information including direct identifiers (contact information) and indirect identifiers (location, occupation, income, etc.), which cannot be publicly shared in accordance with participant consent. Data requests can be sent to the FAMILY project (FAMILY: A Jockey Club Initiative for a Harmonious Society), G/F, Patrick Manson Building, 7 Sassoon Road, Pok Fu Lam, Hong Kong. Email: [jcfamily@hku.hk](mailto:jcfamily@hku.hk)”

R3. We have now added the above data provision statement at the end of the manuscript.

**Reviewers' comments**
Reviewer #2: The author fully addressed reviewers' previous comments and I am impressed. The paper becomes logically complete and the results looks more robust. The paper has more clear discussions on the digital inequality such as the difference among websites, social networks and IM users. I only have a few suggestions before it gets published:

C1. The author's reply about the model selection between Poisson and Logistic is good. It will be good if author add a short footnote about his model selection and the robustness of his Poisson result in the paper.

R1. We notice that footnote is not permitted by PLOS ONE and have added the following: “To test the robustness of results of Poisson regression, ordered logistic regression was used by treating agreement with web-based health information seeking experiences as an ordinal variable (1=very much disagree, 2=somewhat disagree, 3=somewhat agree, 4=very much agree) (S4. File).” in Statistical analyses and “The robustness of results was supported using ordered logistic regression (S4. File).” in Results. Table presenting logistic regression result has been attached as supporting information S4. File.

C2. This research conduct two-stage survey. Could the author briefly explain the reason to do two-stage?

R2. We have now stated in Design and participants as follows: “As we used the same battery of instruments in phase 1 and 2 surveys, datasets were combined to improve the sample size.”

C3. It will be better if the findings can echo some theory of human behavior in the public health field.

R3. We have now stated in Discussion as follows: “The findings supported the “Inverse Care Law” [1], which suggests that the disadvantaged groups are most in need of healthcare but may benefit less from health-related ICTs.”

Reference

1. Hart JT. The inverse care law. The Lancet. 1971;297(7696):405–412.
